# Protective effects of *Carissa opaca* fruits against CCl_4_-induced oxidative kidney lipid peroxidation and trauma in rat

**DOI:** 10.3402/fnr.v59.28438

**Published:** 2015-09-07

**Authors:** Sumaira Sahreen, Muhammad Rashid Khan, Rahmat Ali Khan, Huda Mohammad Alkreathy

**Affiliations:** 1 Botanical Sciences Division, Pakistan Museum of Natural History, Islamabad, Pakistan; 2 Department of Biochemistry, Faculty of Biological Sciences, Quaid-i-Azam University, Islamabad, Pakistan; 3 Department of Biotechnology, University of Science and Technology, Bannu, Pakistan; 4 Department of Pharmacology, Faculty of Medicine, King Abdulaziz University, Jeddah, Saudi Arabia

**Keywords:** *Carissa opaca* fruits, oxidative trauma, genotoxicity, lipid peroxidation, DNA fragmentation

## Abstract

**Background:**

Carbon tetrachloride (CCl_4_) is a potent nephrotoxin, as it causes acute as well as chronic toxicity in kidneys. Therefore, this study was carried out to assess the pharmacological potential of different fractions of *Carissa opaca* fruits on CCl_4_-induced oxidative trauma in the kidney.

**Methods:**

The parameters studied in this respect were the kidney function tests viz, serum profile, urine profile, genotoxicity, characteristic morphological findings, and antioxidant enzymatic level of kidneys.

**Result:**

The protective effects of various fractions of *C. opaca* fruits against CCl_4_ administration were reviewed by rat renal function alterations. Chronic toxicity caused by 8-week treatment of CCl_4_ to the rats significantly decreased the pH level, activities of antioxidant enzymes, and glutathione contents, whereas a significant increase was found in the case of specific gravity, red blood cells, white blood cells, level of urea, and lipid peroxidation in comparison to control group. Administration of various fractions of *C. opaca* fruit with CCl_4_ showed protective ability against CCl_4_ intoxication by restoring the urine profile, activities of antioxidant enzymes, and lipid peroxidation in rat. CCl_4_ induction in rats also caused DNA fragmentation and glomerular atrophy by means of dilation, disappearance of Bowmen's space, congestion in the capillary loops, dilation in renal tubules, and foamy look of epithelial cells of tubular region, which were restored by co-admiration of various fractions of *C. opaca*.

**Conclusion:**

Results revealed that the methanolic fractions of *C. opaca* are the most potent and helpful in kidney trauma.

Carbon tetrachloride (CCl_4_) is a toxic chemical, widely used in the dry cleaning industry, in filling fire extinguishers, in the fumigation of grains, and as an insecticide (1). Recent studies have shown that CCl_4_ is associated with advanced production of free radicals leading to dysfunction of several organs (2). Chronic CCl_4_ treatment is a common practice to induce hepatic fibrosis (3, 4), renal (5, 6), pulmonary (7) and testicular injuries (8), and cardiac tissue damage (9, 10) in rats as an experimental model. Tissue damage by CCl_4_ depends on the amount of dosage and duration of exposure of the experimental animals to this toxicant. Its action is based on membrane lipid peroxidation and induction of trichloromethyl radical (•CCl_3_), resulting in severe cell damage (11). It is evidenced that metabolic activation of CCl_4_ by cytochrome P_450_ resulted in the production of trichloromethyl radical (•CCl_3_) and peroxy trichloromethyl radical (•OOCCl_3_) that, in turn, initiate subsequent lipid peroxidation, responsible for injuries in various organs such as liver and kidney (12–14). Therefore, it can be stated that CCl_4_ is the best-characterized tool for the study of oxidative stress trials as it consistently generates free radicals with the implication of pathological environment. These free radicals damage the integrity of liver cell membranes by releasing the cytosolic enzymes such as alanine transaminase, aspartate transaminase, alkaline phosphatase, and lactate dehydrogenase into the blood stream and elevating thiobarbituric acid reactive substances (TBARS) level with subsequent necrosis and inflammatory cell infiltration; affect physical parameters of kidney such as urinary and serum profile; increase lysosomal enzymes activities of testis and kidney; and decrease the activity of a diagnostic marker enzyme creatinine phosphokinase (an enzyme responsible for ATP regeneration) in cardiac tissue (15). The health-promoting effect of antioxidants on oxidative damage is mostly examined through the cellular antioxidants including the enzymatic behavior of catalase (CAT), peroxidase (POD), superoxide dismutase (SOD), glutathione peroxidase (GPx), glutathione-S-transferase (GST), and glutathione reductase (GR), in addition to TBARS (a product of lipid peroxidation) and reduced glutathione (GSH) level (16) among various tissues. Previous studies have shown that antioxidants, including naringenin, N-acetyl cysteine, vitamin E, silymarin, quercetin, and rhein decrease lipid peroxidation, and partially ameliorate tissue injuries (16). Over the years, many researchers have reported that plants containing phenolics and flavonoids exhibit a large array of biological activities such as hepatoprotection, cardio-protection, and reversal of fibrosis. These phytochemicals are widely found in fruits and vegetables as an important part of the human diet. Consequently, plants like dietary substances may be reliable guards against tissue damage, as well as a mechanism in curtailing the progression to lethal diseases like cancer (17). *Carissa opaca* Stapf ex Hanes is a 2–3 m tall evergreen shrub containing glabrous or puberulous branches with opposite and ovate glabrous leaves, hard and sharp spines arising between the petiole widely found in Pakistan and in some areas of India, Burma, and Sri Lanka (17). Traditionally, this plant is used for the treatment of asthma and pulmonary diseases (18), anticancer (19), diarrhea (20), hepatoprotection (21), and reproductive dysfunction (22). Therefore, the present study aimed to assess the nephro-protective potential of different fractions of *C. opaca* fruits on CCl_4_-induced oxidative trauma in the kidney.

## Plant collection

The ripened fruits of *C. opaca* were collected in March–April 2011 from the Quaid-i-Azam University Islamabad and Abbottabad of Northern Pakistan, respectively. The plants were recognized by their local names and then validated by Dr. Mir Ajab Khan, Department of Plant Sciences, Quaid-i-Azam University, Islamabad, and Dr. Saleem Ahmad, Curator, Pakistan Museum of Natural History, Islamabad. A voucher specimen with Accession No. 24561 (*C. opaca*) was deposited at the Herbarium of Pakistan, Museum of Natural History, Islamabad.

## Extract preparation

The collected plant samples were cleaned to get rid of dust particles and then dried under shade for 1–2 weeks. Willy Mill of 60-mesh size was used to prepare powder of dried samples, and then each powdered plant sample was used for further solvent extraction. First of all, 5 kg of powdered sample was extracted twice with 10 L of 95% methanol at 25°C for 48 h. For filtration, Whatman No. 1 filter paper was used, and then the filtrate was concentrated on rotary evaporator (Panchun Scientific Co., Kaohsiung, Taiwan) under reduced pressure at 40°C. To resolve the compounds with escalating polarity, a part of the extract was suspended in distilled water and subjected to liquid–liquid partition by using solvents in the sequence of n-hexane, ethyl acetate, and methanol. After fractioning, the solvent of respective fractions was also evaporated by rotary evaporator. Fractions of n-hexane (HFC), ethyl acetate (EFC), and methanol (MFC) were obtained and were stored at 4°C for further *in vivo* investigation.

## Experimental plan

A total of 42, 6-week-old, male Sprague–Dawley rats weighing 180±10 g were purchased from the National Institute of Health (NIH), Islamabad. Rats were given food and water *ad libitum* and kept at 20–22°C on a 12-h light–dark cycle. The study protocol was approved by ethical committee of Quaid-i-Azam University, Islamabad. The rats were acclimatized to laboratory condition for 7 days before commencement of experiments and then randomly divided into seven groups (six rats per group). Administration of CCl_4_ (0.5 ml/kg b.w., 20% CCl_4_/olive oil) was intraperitoneally (i.p.) administered twice a week for 8 weeks. At the same time, the rats were individually administered silymarin (50 mg/kg b.w.) in DMSO and various fractions including HFC, EFC, and MFC (200 mg/kg b.w.) orally twice a week for 8 weeks. At the end of 8 weeks, 24 h after the last treatment, urine was collected and stored at −70°C for further analysis, and then animals were given chloroform anesthesia and then dissected. All animals were sacrificed; blood was drawn prior to the excision of organ tissues from the ventral side through cardiac puncture. The serum was stored at −80°C after separation until it was assayed, as described below. Kidneys were removed and washed in ice-cold saline. Subsequently, half of the organs were treated with liquid nitrogen and stored at −80°C for further enzymatic and DNA damage analysis while the other portion was processed for histology (5).

## Biochemical investigations

Evaluation of the pharmacological effects of different fractions of *C. opaca* against the toxicity induced with CCl_4_ in rats has been done following assays as described by Khan et al. (5).

### Physical analysis of urine

Urine samples were assayed for red blood cells (RBCs) count, white blood cells (WBCs) count, urobilinogene, pH, and specific gravity by using standard diagnostic kits (MediScreen Urine Strips, Orgenics, France).

### Biochemical analysis of urine and serum

Estimation of total protein, albumin, urea, creatinine, and creatinine clearance was done by using standard diagnostic kits (MediScreen kit France).

### Assessment of antioxidant enzymes

In total, 10% homogenate of tissue was prepared in 100 mM KH_2_PO_4_ buffer containing 1 mM ethylenediaminetetraacetic acid (EDTA; pH 7.4) and centrifuged at 12,000×*g* for 30 min at 4°C. The supernatant was collected and used for the following parameters as described below.

#### Catalase assay

CAT activities were determined by the method of Chance and Maehly (23) with some modifications. The reaction solution of CAT activities contained: 2.5 ml of 50 mM phosphate buffer (pH 5.0), 0.4 ml of 5.9 mM H_2_O_2_, and 0.1 ml enzyme extract of 10% (100 mg) tissue homogenate. Changes in absorbance of the reaction solution at 240 nm were determined after 1 min. One unit of CAT activity was defined as an absorbance change of 0.01 as units/min.

#### Peroxidase assay

Activities of POD were determined by the method of Chance and Maehly (23) with some modifications. The POD reaction solution contained: 2.5 ml of 50 mM phosphate buffer (pH 5.0), 0.1 ml of 20 mM guaiacol, 0.3 ml of 40 mM H_2_O_2_, and 0.1 ml enzyme extract. Changes in absorbance of the reaction solution at 470 nm were determined after 1 min. One unit of POD activity was defined as an absorbance change of 0.01 units/min.

#### Superoxide dismutase assay

SOD activity was estimated by the method of Kakkar et al. (24). A mixture containing 0.1 ml of phenazine methosulfate (186 µM), 1.2 ml of sodium pyrophosphate buffer (0.052 mM; pH 7.0), and 0.3 ml of supernatant after centrifugation (1,500×g for 10 min followed by 10,000×g for 15 min) of kidney homogenatewasadded to this reaction mixture. Enzyme reaction was initiated by adding 0.2 ml of NADH (780 µM) and stopped after 1 min by adding 1 ml of glacial acetic acid. Amount of chromogen formed was measured by recording color intensity at 560 nm. Results are expressed in units/mg protein.

#### Glutathione-S-transferase assay

GST activity was assayed by the method of Habig et al. (25). The reaction mixture consisted of 1.475 ml phosphate buffer (0.1 mol, pH 6.5), 0.2 ml reduced glutathione (1 mM), 0.025 ml (CDNB) (1 mM), and 0.3 ml of homogenate in a total volume of 2.0 ml. The changes in the absorbance were recorded at 340 nm, and enzymes activity was calculated as nM CDNB conjugate formed/min/mg protein using a molar extinction coefficient of 9.6×10^3^ M^−1^cm^−1^.

#### Glutathione reductase assay

GR activity was determined by the method of Carlberg and Mannervik (26). The reaction mixture consisted of 1.65 ml phosphate buffer: (0.1 mol; pH 7.6), 0.1 ml (0.5 mM EDTA), 0.05 ml oxidized glutathione (1 mM), 0.1 ml nicotinamide adenine dinucleotide phosphate (NADPH), and 0.1 ml of homogenate in a total volume of 2 ml. Enzyme activity was quantitated at 25°C by measuring the disappearance of NADPH at 340 nm and was calculated as nM NADPH oxidized/min/mg protein using molar extinction coefficient of 6.22×10^3^ M^−1^cm^−1^.

#### Glutathione peroxidase assay

GPx activity was assayed by the method of Mohandas et al. (27). The reaction mixture consisted of 1.49 ml phosphate buffer (0.1 M; pH 7.4), 0.1 ml EDTA (1 mM), 0.1 ml sodium azide (1 mM), 0.05 ml GR (1 IU/ml), 0.05 ml GSH (1 mM), 0.1 ml NADPH (0.2 mM), 0.01 ml H_2_O_2_ (0.25 mM), and 0.1 ml of homogenate in a total volume of 2 ml. The disappearance of NADPH at 340 nm was recorded at 25°C. Enzyme activity was calculated as nM NADPH oxidized/min/mg protein using molar extinction coefficient of 6.22×10^3^ M^−1^cm^−1^.

#### Quinone reductase assay

The activity of quinone reductase (QR) was determined by the method of Benson et al. (28). The 3.0 ml reaction mixture consisted of 2.13 ml Tris-HCl buffer (25 mM; pH 7.4), 0.7 ml bovine serum albumin, 0.1 ml FAD, 0.02 ml NADPH (0.1 mM), and 0.l ml of homogenate. The reduction of dichlorophenolindophenol (DCPIP) was recorded at 600 nm, and enzyme activity was calculated as nM of DCPIP reduced/min/mg protein using molar extinction coefficient of 2.1×10^4^ M^−1^cm^−1^.

#### Reduced glutathione assay

Reduced GSH was estimated by the method of Jollow et al. (29). A volume of 1.0 ml of homogenate was precipitated with 1.0 ml of (4%) sulfosalicylic acid. The samples were kept at 4°C for 1 h and then centrifuged at 1,200×g for 20 min at 4°C. The total volume of 3.0 ml assay mixture contained 0.1 ml filtered aliquot, 2.7 ml phosphate buffer (0.1 M; pH 7.4), and 0.2 ml DTNB (100 mM). The yellow color developed was read immediately at 412 nm on a SmartSpecTM plus Spectrophotometer. It was expressed as µM GSH/g tissue.

#### Estimation of lipid peroxidation assay

The assay for lipid peroxidation was carried out following the modified method of Iqbal et al. (30). The reaction mixture in a total volume of 1.0 ml contained 0.58 ml phosphate buffer (0.1 M; pH 7.4), 0.2 ml homogenate sample, 0.2 ml ascorbic acid (100 mM), and 0.02 ml ferric chloride (100 mM). The reaction mixture was incubated at 37°C in a shaking water bath for 1 h. The reaction was stopped by the addition of 1.0 ml of 10% trichloroacetic acid (TCA). Following the addition of 1.0 ml of 0.67% thiobarbituric acid, all the tubes were placed in a boiling water bath for 20 min and then shifted to a crushed ice-bath before centrifuging at 2,500×*g* for 10 min. The amount of TBARS formed in each of the samples was assessed by measuring the optical density of the supernatant at 535 nm using spectrophotometer against a reagent blank. The results were expressed as nM TBARS/min/mg tissue at 37°C using molar extinction coefficient of 1.56 ×10^5^ M^−1^cm^−1^.

#### Hydrogen peroxide assay

Hydrogen peroxide (H_2_O_2_) was assayed by H_2_O_2_-mediated horseradish peroxidase-dependent oxidation of phenol red by the method of Pick and Keisari (31). A volume of 2.0 ml of homogenate sample was suspended in 1.0 ml of solution containing phenol red (0.28 nM), horse radish peroxidase (8.5 units), dextrose (5.5 nM), and phosphate buffer (0.05 M; pH 7.0) and incubated at 37°C for 60 min. The reaction was stopped by the addition of 0.01 ml of NaOH (10 N) and then centrifuged at 800×*g* for 5 min. The absorbance of the supernatant was recorded at 610 nm against a reagent blank. The quantity of H_2_O_2_ produced was expressed as nM H_2_O_2_/min/mg tissue based on the standard curve of H_2_O_2_ oxidized phenol red.

### Molecular studies

DNA has been isolated and its fragmentation percent was quantified in molecular studies of *in vivo* toxicity.

#### DNA fragmentation assay with diphenylamine reaction

DNA fragmentation from tissue extract was determined using the procedure of Wu et al. (32). A quantity of 100 mg tissue was homogenized in Tris triton EDTA (TTE) solution. A volume of 0.1 ml of homogenate was labeled B, centrifuged at 200×*g* at 4°C for 10 min, and the supernatant was collected and labeled S. S tubes were centrifuged at 20,000×*g* for 10 min at 4°C to separate intact chromatin and was labeled T. A volume of 1.0 ml of 25% TCA was added to all tubes T, B, S and incubated overnight at 4°C. After incubation, precipitated DNA was recovered by pelleting for 10 min at 18,000×*g* at 4°C. A volume of 160 µl of 5% TCA was added to each pellet and heated for 15 min at 90°C, then 320 µl of freshly prepared DPA solution was added, vortexed, and incubated for 4 h 37°C. Optical density was read at 600 nm with a spectrophotometer (Smart spec™ Plus, catalogue # 170-2525).

#### DNA isolations and ladder assay

DNA was isolated by using the methods of Wu et al. (32). A quantity of 100 mg of tissue in a Petri dish was washed with DNA buffer and homogenized in 1 ml of lysis buffer. A volume of 100 µl of proteinase K (10 mg/ml) and 240 µl of 10% SDS were added shaken gently, and incubated overnight at 45°C in a water bath, then 0.4 ml of phenol was added and shaken for 5–10 min and centrifuged at 3,000 rpm for 5 min at 10°C. Supernatant was mixed with 1.2 ml of phenol, 1.2 ml of chloroform/isoamyl alcohol (24:1); shaken for 5–10 min and centrifuged at 3,000 rpm for 5 min at 10°C. A volume of 25 µl of 3 M sodium acetate (pH 5.2) and 5 ml ethanol was added with supernatant, shaken until DNA was precipitated. DNA was washed with 70% ethanol, dried, dissolved in TE buffer and its concentration checked at 260 and 280 nm. A quantity of 5 µg of total DNA and 0.5 µg of DNA standard per well were loaded on 1.5% agarose gel containing ethidium bromide. Electrophoresis was performed for 45 min with 100 V batteries, and DNA was observed under digital gel doc system and photographed.

### Histopathological study of tissue

After weighting the portion specified for histology, small pieces of each tissue were fixed for 3–4 h in fixative sera followed by dehydration with ascending grades of alcohol (80, 90, and 100%) and transferred in cedar wood oil. When tissue became clear, then all tissues were embedded in paraplast and prepared blocks for further microtomy. Thin slides of 3–4 µm were prepared with microtome; wax was removed, stained with hematoxylin–eosin and photographed under a light microscope at 10× and 40×.

### Statistical analysis

To find the different treatment effects of *in vivo* studies, one-way analysis of variance was carried out by using the computer software SPSS 13.0. The level of significance among the various treatments was determined by LSD at 0.05% level of probability.

## Results

### Effects of C. opaca fruit against CCl_4_-induced nephrotoxicity in rat

The present achievements were used to assess the medicinal potential of various fractions of *C. opaca* fruit against CCl_4_-induced oxidative damages in rat kidney. The biochemical alterations in different groups of rats were collaborated by the renal histological architectures of respective groups. The results obtained in this context are given below.

### Effects of C. opaca fruits on urine profile of rat

The protective effects of various fractions of *C. opaca* fruits against CCl_4_ administration were reviewed by rat renal functions alterations. Table 1 presents urine profile including urinary pH, specific gravity, RBCs, WBCs, and level of urea. Chronic toxicity caused by 8-week treatment of CCl_4_ to the rats significantly decreased the pH level, whereas a significant increase was found in the cases of specific gravity, RBCs, WBCs, and level of urea in comparison to control group. Table 2 demonstrates a marked increase in urinary creatinine and urobilinogen whereas creatinine clearance, urinary albumin, and level of urinary proteins were significantly decreased by CCl_4_ administration (*p*<0.05) in contrast to control group. Administration of various fractions of *C. opaca* fruit with CCl_4_ showed protective ability against CCl_4_ intoxication by restoring the urine levels of rat. Results of urine analysis for the groups treated with fractions alone did not show any toxic effects as their values were near to control. Data show that almost all fractions had significant pharmacological effects for urine versus diseased group.

**Table 1:** Effects of various fractions of *C. opaca* fruits on urine profile including pH, specific gravity, RBC, WBC, and urea

Group	pH	Specific gravity	RBC/µl	WBC/µl	Urea (mg/dl)
Control	7.01±0.00d	1.04±0.02c	0.01±0.01d	30.72±2.34f	72.23±2.21e
Oil+DMSO	7.03±0.04d	1.06±0.04c	0.00±0.00d	32.37±1.46f	69.18±2.10e
CCl_4_	6.20±0.01a	1.40±0.05a	15.4±2.6a	93.78±3.34a	99.31±3.42a
Sily+CCl_4_	6.80±0.07c	1.10±0.02c	4.65±0.17e	46.12±2.57e	78.03±1.85d
HFC+CCl_4_	6.34±0.12b	1.28±0.04b	11.65±0.49b	80.18±2.37b	92.34±1.26b
EFC+CCl_4_	6.40±0.10b	1.20±0.06b	10.27±0.66b	77.12±2.37b	93.26±1.62b
MFC+CCl_4_	6.64±0.09c	1.18±0.05b	8.48±0.61c	56.27±3.28d	85.28±1.48c

Values are mean±SD (06 number). Sily, silymarin.

Means with different letters indicate significance at *p*<0.05.

**Table 2:** Effects of various fractions of *C. opaca* fruits on urine profile viz, creatinine, creatinine clearance, albumin, urobilinogen, and urinary proteins

Group	Creatinine (mg/dl)	Creatinine clearance (ml/min)	Albumin (mg/dl)	Urobilinogen (mg/dl)	Urinary protein (mg/dl)
Control	2.04±0.22c	1.63±0.04d	9.67±0.26e	4.42±1.31f	30.42±0.19f
Oil+DMSO	2.00+0.10c	1.59±0.01d	9.41±0.71e	4.45±1.03f	29.19±0.26f
CCl_4_	4.67±1.26a	0.85±0.02a	4.06±0.12a	36.14±1.26a	15.79±0.74a
Sily+CCl_4_	2.77±0.32b	1.40±0.06c	7.70±0.25d	10.21±1.78e	26.21±0.36e
HFC+CCl_4_	3.00±0.65b	1.00±0.07b	5.10±0.23b	31.27±2.19b	17.11±0.42b
EFC+CCl_4_	3.07±0.44b	1.03±0.05b	5.31±0.30b	29.16±2.49b	18.31±0.51b
MFC+CCl_4_	3.04±0.23b	1.24±0.09c	6.56±0.22c	25.25±2.65c	22.50±0.28d

Values are mean±SD (06 number). Sily, silymarin.

Means with different letters indicate significance at *p*<0.05.

### Effects of C. opaca fruits on serum profile of rats

Effects of different fractions of *C. opaca* fruits on serum profile of kidneys are summarized in Tables 3 and 4. Table 3 presents serum protein, albumin, globulin, total bilirubin, and direct bilirubin of serum. There was a significant reduction in protein, albumin, and globulin level of serum in CCl_4_-toxicated group, while total bilirubin and direct bilirubin of serum were improved in CCl_4_-toxicated group. Table 4 summarizes the blood urea nitrogen (BUN), urobilinogen, creatinine, creatinine clearance, and nitrite level of serum. Administration of CCl_4_ significantly enhanced the BUN, urobilinogen, creatinine, and nitrite level of serum, whereas reduced the creatinine clearance of serum. Silymarin was used as a reference drug to reduce nephrotoxicity.

**Table 3:** Effects of various fractions of *C. opaca* fruits on serum profile such as serum proteins, albumin, globulin, total bilirubin, and direct bilirubin

Group	Serum proteins (mg/dl)	Albumin (mg/dl)	Globulin (mg/dl)	Total bilirubin (mg/dl)	Direct bilirubin (mg/dl)
Control	45.31±0.47d	20.0±0.28e	39.27±1.23e	3.09±0.07d	1.00±0.06d
Oil+DMSO	47.11±0.60d	19.63±0.47e	38.01±1.75e	3.00±0.10d	1.08±0.03d
CCl_4_	29.79±0.83a	10.38±0.52a	24.19±0.39a	4.34±0.29a	1.86±0.13a
Sily+CCl_4_	37.26±0.48c	17.74±0.36d	32.22±1.20d	3.28±0.09c	1.21±0.07c
HFC+CCl_4_	34.45±0.57b	11.56±0.35b	26.21±0.62b	4.05±0.07a	1.65±0.05b
EFC+CCl_4_	33.45±0.63b	11.87±0.31b	26.11±0.28b	3.98±0.04a	1.62±0.04b
MFC+CCl_4_	32.24±0.56b	14.34±0.33c	28.37±1.07c	3.62±0.12b	1.45±0.10c

Values are mean±SD (06 number). Sily, silymarin.

Means with different letters indicate significance at *p*<0.05.

**Table 4:** Effects of various fractions of *C. opaca* fruits on urine profile including BUN, urobilinogen, creatinine, creatinine clearance, and serum nitrite

Group	BUN (mg/dl)	Urobilinogen (mg/dl)	Creatinine (mg/dl)	Creatinine clearance (ml/min)	Serum nitrite (µM/ml)
Control	32.27±1.28e	11.01±2.01e	45.00±2.11d	0.98±0.00c	42.34±1.24e
Oil+DMSO	33.11±1.10e	10.23±1.92e	44.21±1.54d	0.93±0.04c	44.21±1.43e
CCl_4_	62.28±2.19a	40.21±3.10a	72.75±3.45a	0.63±0.03a	81.34±3.45a
Sily+CCl_4_	41.71±1.63d	19.30±2.18d	52.15±2.03c	0.84±0.03b	53.34±2.39d
HFC+CCl_4_	58.44±1.27b	34.20±1.26b	65.45±2.19b	0.68±0.05a	75.30±1.57b
EFC+CCl_4_	57.50±1.68b	33.16±2.27b	63.47±2.33b	0.69±0.03a	74.44±1.64b
MFC+CCl_4_	51.27±1.73c	27.29±1.19c	58.33±1.56c	0.74±0.05b	63.50±2.52c

Values are mean±SD (06 number). Sily, silymarin.

Means with different letters indicate significance at *p*<0.05.

### Effects of C. opaca fruits on antioxidant levels

Nature has developed metabolic systems in various organisms to reduce the oxidative damage and avoid different lethal damage to cells and tissues. During this natural process, various oxidative metabolites are formed as a by-product in the cells. Antioxidant enzymes reduce these metabolites in a scavenging process. In this study, activities and scavenging effects of various antioxidative enzymes were studied. Effects of various fractions of *C. opaca* fruits and CCl_4_ on tissue soluble protein and antioxidant defense enzyme systems such as CAT, POD, SOD, TBARS, and H_2_O_2_ are shown in Table 5. CCl_4_ administration to rats significantly decreased tissue protein and disturbs the antioxidative status by lessening the renal catalase, peroxidase, and superoxidase levels, and increased the lipid peroxidation and hydrogen peroxide levels in comparison to control group. Co-administration of various fractions of *C. opaca* fruits with CCl_4_ enhanced the activity of suppressed enzymes and the soluble protein, whereas reduced the activity of elevated enzymes. However, non-significant change was recorded with the treatment of fractions alone against the control group.

**Table 5:** Effects of various fractions of *C. opaca* fruits on tissue proteins and antioxidant enzyme levels

Group	Protein (µg/mg tissue)	CAT (U/min)	POD (U/min)	SOD (U/mg protein)	TBARS (nM/min/mg protein)	H_2_O_2_ (µM/ml)
Control	2.20±0.061e	5.04±0.32d	12.35±0.18e	3.44±0.20e	2.97±0.08e	1.31±0.012e
Oil+DMSO	2.21±0.039e	4.96±0.48d	12.31±0.21e	3.38±0.23e	2.95±0.13e	1.25±0.013e
CCl_4_	0.93±0.019a	2.50±0.18a	6.00±0.32a	1.00±0.08a	6.07±0.66a	2.98±0.019a
Sily+CCl_4_	1.83±0.038d	4.02±0.39c	10.21±0.41d	2.78±0.33d	3.91±0.17d	1.76±0.031d
HFC+CCl_4_	1.21±0.033b	3.04±0.38b	7.79±0.22b	1.58±0.12b	5.41±0.11b	2.54±0.064b
EFC+CCl_4_	1.19±0.047b	2.99±0.31b	7.76±0.23b	1.69±0.21b	5.34±0.13b	2.51±0.065b
MFC+CCl_4_	1.54±0.028c	3.41±0.39b	8.80±0.42c	2.20±0.31c	4.02±0.23c	1.89±0.074c

Values are mean±SD (06 number). Sily, silymarin.

Means with different letters indicate significance at *p*<0.05.

Table 6 shows the levels of phase II metabolizing antioxidant enzymes including GST, GPx, GR, GSH, QR, and DNA fragmentation in renal tissues of different experimental groups. CCl_4_ insults extensively (*p*<0.05) lessened the levels of GST, GPx, GR, GSH, and QR while the DNA fragmentation was enhanced. Post-administration of various fractions of *C. opaca* fruits along with CCl_4_ treatment markedly reversed the level of GST, GPx, GR, GSH, QR, and DNA fragmentation. Silymarin administered to the intoxicated group completely suppressed the effect of CCl_4_ and increased the level of glutathione enzymatic group but decreased the level of DNA injuries. Treatment of rats with CCl_4_ significantly increased the renal tissue DNA damages than those of control group. The groups treated with various fractions of *C. opaca* fruits administered with CCl_4_ significantly abridged the DNA fragmentation versus the diseased group treated with CCl_4_ alone. The results of oxidative enzymatic studies are also in harmony with the consequences of urinary and serological studies.

**Table 6:** Effects of various fractions of *C. opaca* fruits on phase II antioxidant enzymes and DNA fragmentation

Group	GST (nM/mg protein)	GPx (nM/mg protein)	GR (nM/mg protein)	GSH (µM/tissue)	QR (nM/mg protein)	% DNA injuries
Control	190.5±6.3f	130.6±3.3d	218.2±7.38e	20.9±1.47d	100.28±2.3d	21.72±1.34e
Oil+DMSO	193.8±6.2f	126.41±3.8d	209.46±6.27e	24.64±1.34d	107.34±2.4d	20.13±1.44e
CCl_4_	95.92±3.1a	75.28±3.1a	117.23±4.64a	10.13±0.83a	65.44±2.56a	57.70±2.24a
Sily+CCl_4_	155.62±3.8d	112.28±2.7c	195.67±4.58d	17.44±0.91c	94.36±2.41c	25.40±1.37d
HFC+CCl_4_	112.3±1.1b	83.11±1.8a	150.32±3.56b	12.35±0.46b	70.34±1.35b	33.43±0.64b
EFC+CCl_4_	114.6±2.0b	84.37±1.7a	152.35±2.41b	11.77±0.52b	71.11±1.20b	33.27±0.45b
MFC+CCl_4_	130.3±2.5c	100.6±2.78b	190.86±3.99c	16.06±1.00c	90.25±2.49c	30.15±1.31c

Values are mean±SD (06 number). Sily, silymarin.

Means with different letters indicate significance at *p*<0.05.

### Effects of C. opaca fruits on DNA damages (ladder assay)

**Fig. 1:**
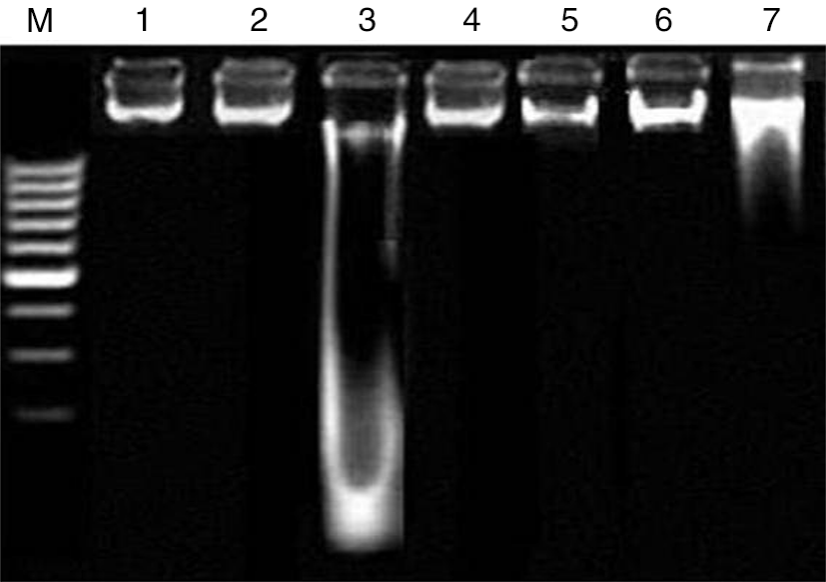
Agarose gel showing DNA damage by CCl_4_ and protective effects of various fractions of *C. opaca* fruits in renal tissue. Lanes from left: (M) low molecular weight marker, (1) control, (2) DMSO+Olive oil group, (3) CCl_4_ group, (4) silymarin+CCl_4_ group, (5) MFC+CCl_4_ group, (6) EFC+CCl_4_ group, (7) HFC+CCl_4_ group.

DNA was extracted from renal tissues of the treated rats by a stepwise method and then was loaded on 1.5% agarose gel. Figure 1 details the banding pattern of DNA. In case of CCl_4_-intoxicated rats, a peculiar fragmentation of DNA can be observed, which was absent from the renal tissues of control rats. DNA extracted from the group treated with various fractions of *C. opaca* fruits along with CCl_4_ showed a markedly repaired DNA. The groups treated with only various fractions did not show any kind of DNA damage.

### Effects of C. opaca fruits on kidney histoarchitecture

**Fig. 2:**
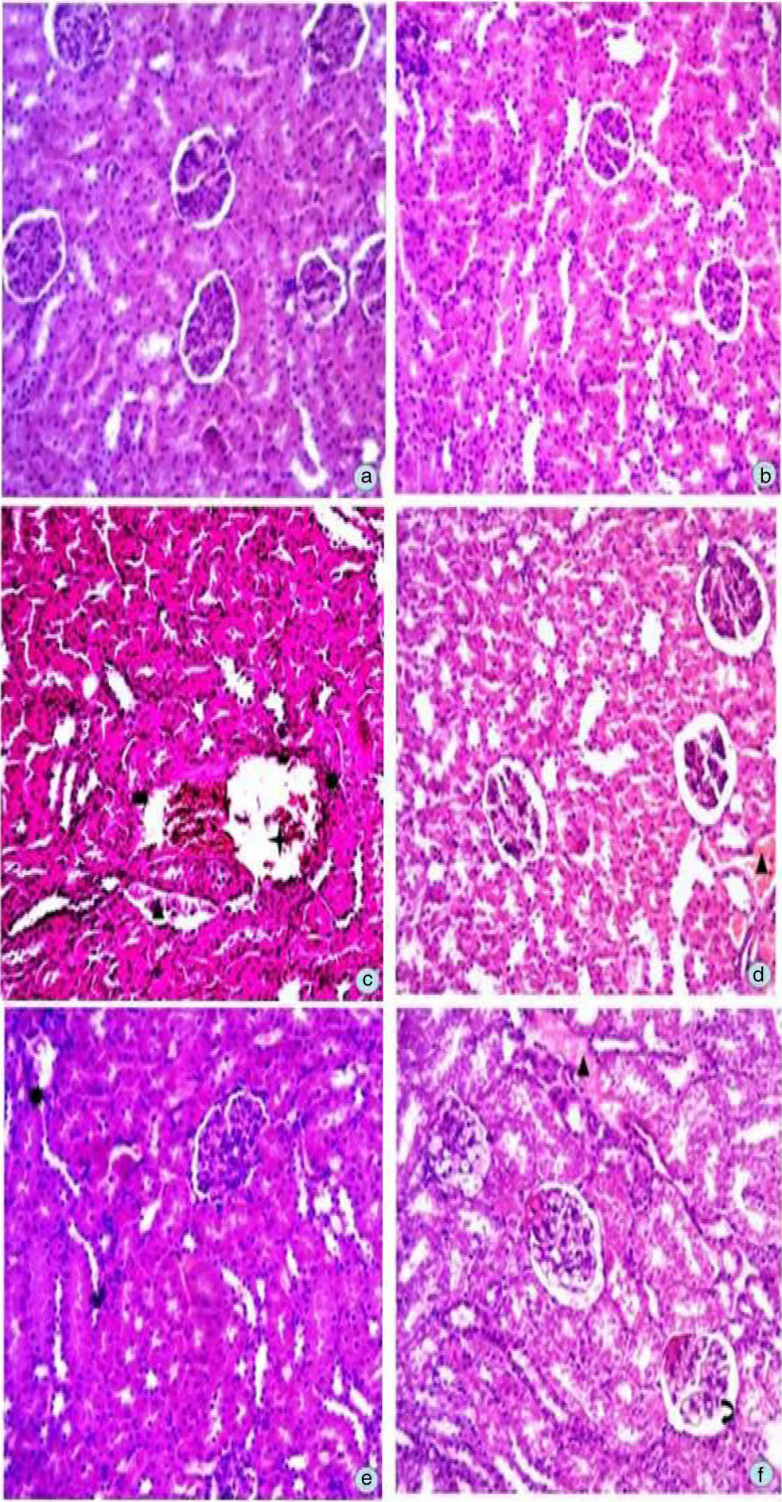
Microphotograph of rat kidney (hematoxylin and eosin stain) (a). Representative section of renal from the control group showing normal histology, (b) DMSO+Olive oil group, (c) CCl_4_ group, (d) silymarin+CCl_4_ group, (e) MFC+CCl_4_ group, (f) EFC+CCl_4_ group, (g) HFC+CCl_4_ group, (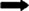) glomerular atrophy, (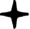) vascular congestions around the tubules, (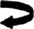) alterations in Bowmen's space, (▲) tubular dilations, (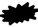) distorted renal corpuscles.

Histoarchitecture of kidneys of different groups is summarized in Fig. 2. The histopathologically observed changes showed that the intraperitoneal injection of CCl_4_ for 8 weeks resulted in severe impairment to corticular region of kidneys. The damage induced was in different forms of degenerations showing glomerular atrophy by means of dilation and disappearance of Bowmen's space, congestion in the capillary loops, dilation in renal tubules, and foamy look of epithelial cells of tubular region (Fig. 2c). In addition, subchronic administration of CCl_4_ exhibited interstitial inflammatory cells infiltration in both corticular and medullary regions. The evaluation of kidney section of control and DMSO group had normal histological architecture as shown in Fig. 2a and b. Renal sections of rats treated with various fractions of *C. opaca* fruits (HFC, EFC, and MFC) reversed the CCl_4_ intoxication and showed mild injury in contrast to control group (Fig. 2d–f). The histological appearance of glomeruli and Bowman's capsule was almost normal, and only some of the glomeruli were degenerated. Mild necrosis in the glomeruli and tubules was observed. As a measure of protective effects, silymarin was also induced to a group of rats (Fig. 2d). Silymarin treatment erased the CCl_4_ pathogenesis, relative to control group. These findings are in agreement with the rest of the parameters studied.

## Discussion

As we previously investigated, the methanol fraction of *C. opaca* is rich in active compounds such as orientin, isoquercetin, myricetin, and apigenin (20). Hence, it is proposed that these fractions might have protection against degenerative diseases caused by oxidative stress. CCl_4_ intoxication generates free radicals that trigger a cascade of events resulting in nephrotoxicity. Activation of cytochrome P_450_ oxygenase system yields highly reactive trichloromethyl peroxy radical that initiate lipid peroxidation (33). The lipid peroxidation may cause peroxidative tissue damage (34). Relevant to the latter findings, plant extract and its fractions comprehensively ameliorate the injuries induced through CCl_4_ intoxication and affect the activity of biochemical enzymes, DNA strand breakage, and increase the activity of telomerase cancer marker enzyme of kidney tissue (35). The protective effects might seem to be related to their molecular structure, more precisely to the presence and number of hydroxyl groups, and to double bond conjugation and resonance effects substituents in the phenyl ring and on the position of its nitrogen atom relative to the N–H bond as was previously reported (36, 37).

Previous studies reported that the urine analysis provides information about the functional status of kidneys. It can be said that malfunctioning kidneys result in distressed urine profiles. Urobilinogen, RBCs, and acidic pH are not the components of urine in normal conditions; however, the presence of significantly higher amount of urobilinogen, RBCs, and acidic pH was observed in urine that may be the result of oxidative damage in kidney of CCl_4_-intoxicated rats. Besides this, the elevated levels of WBCs, urinary creatinine, and urea, and decline in creatinine clearance, albumin, and proteins of urine were an indication of severe kidney damage. Similarly, the specific gravity of urine correlates with osmolality (36), and increased specific gravity is one of the symptoms of kidney damages such as dehydration, renal artery steatosis, necrosis, decreased blood flow to kidneys, and proteinuria (37). The present study exposed that various fractions of tested plant samples showed different levels of ameliorating effects by normalization of urinary profile.

Sedlak and Snyder (38) reported that the bilirubin is an important physiological cytoprotectant due to its antioxidant ability. At present, administration of CCl_4_ indicated the risk of impaired-renal function by elevation of serum creatinine, BUN, nitrite level, urobilinogen, total bilirubin, and direct bilirubin (39). Decrease in total globulin and albumin of serum pointed toward leakage due to injuries in the kidney. Decrease in serum creatinine, BUN, nitrite level, urobilinogen, total and direct bilirubin concentrations of groups that were treated with different plant fractions confirms a contributory mechanism of reduced oxidative stress. Previous studies have also revealed the similar conclusions against CCl_4_-induced oxidative stress in kidneys while dealing with different plant extracts/fractions or bioflavonoids (40). But our results discord as well as confirm the findings of Ogeturk et al. (41) who reported non-significant change but increase in serum creatinine level after 1 month of CCl_4_ intoxication. Confliction may be attributed to lesser period of dosage administration. Urinary and serum profile studies proved their concomitance with renal functionality. CCl_4_ intoxication alters the gene expression level by depleting renal SOD and catalase (42) while our tested fractions of plant samples ameliorated the renal toxicity by alleviated level of catalase, peroxidase, and SOD. GSH acts as a non-enzymatic protein thiol contributing in defense system against oxidative damages of both intracellular and extracellular environment. It participates in various enzymatic processes to reduce the concentration of peroxides by its redox and detoxification reaction (43). Under oxidative stress conditions reduced GSH is oxidized, thus it specifies that depletion in GSH content and an increase in TBARS and H_2_O_2_ of kidney tissue are an indication of strong renal injuries. Administration of different fractions of tested plant samples showed a clear-cut elevation in the amount of GSH that was concomitant with a reduction in TBARS and H_2_O_2_ contents, hence verifying the protection against CCl_4_ challenges. These findings were consistent with the literature. Our results are consistent with Soni et al. (44) who reported that CCl_4_-induced oxidation caused an increase in TBARS content and lessened antioxidant enzymatic and non-enzymatic indicators of serum and renal tissue. Present observations are in agreement with previous demonstrations that hesperidin is a bioflavonoid, which reduces the production of CCl_4_-induced free radical by improving GSH levels of kidneys and decreases the concentration of 8-hydroxydeoxyguanosine (8-OH-dG), a marker of DNA fragmentation (45). Consequently, improvement in antioxidant enzyme levels such as CAT, POD, SOD, and phase II metabolizing enzymes (GST, GR, GPx, and QR) in contrast to CCl_4_-injected rats revealed the antioxidative trend of different fractions of tested plant samples. Increased DNA damage ultimately turns into cell proliferation and apoptosis (46–48). CCl_4_ administration is the major cause of oxidative stress and reactive oxygen species in the cell. It enhances the formation of the lipid peroxide radicals, which accelerate the breakdown of polyunsaturated fatty acids (PUFAs). Persistent oxidative stress induces DNA damage by modifying base products and results in strand breaks, which may lead to further mutation and chromosomal aberrations (49). Endonucleases degrade the cellular DNA to induce tubular epithelial cell death of kidney tissue. Overproduction of free radicals triggers the activity of Deoxyribonuclease I (DNase I) (50). DNase I, a highly reactive renal endonuclease I, attributes to about 80% of the total endonuclease action in renal tissues (51). Under these circumstances, it is possible that prevention of CCl_4_-induced renal DNA damage might be due to modulation of DNase I activity. Our findings are consistent with Nagwani and Tripathi (52), who reported similar results in amelioration of cisplatin-induced nephrotoxicity by PTY, a herbal preparation. The histopathological findings in kidney correlate with biochemical estimations of studied experimental groups. CCl_4_ challenges produced an ischemic environment leading to glomerular and tubular lesions with vasocongestion in kidney. Similar histopathological architecture was observed by Dogukan et al. (53) in renal tissue of rats in response to chronic administration of CCl_4_ for 7 weeks. It is also thought that histopathological alterations may be associated with the absorption power of renal tubules that introduce functional overloading of nephrons with subsequent renal dysfunction (12). Regarding other kidney function tests, it can be revealed that the mechanism of CCl_4_ to intend nephrotoxicity and hepatotoxicity is probably the same. It can be concluded that the natural antioxidants such as flavonoids or plant fractions are direct means to protect the cell against potent toxins and chemicals; hence, the present study verifies the enhanced repairing effects of different fractions of tested plant materials. Our findings are in harmony with other studies who reported that different fractions of plants have pharmacological effects by erasing the CCl_4_ insults and reverting toward normalcy (41).

## Conclusion

The present study provides additional scientific evidences verifying that methanol extracts of *C. opaca* are strong antioxidants capable of protecting the kidney from CCl_4_-induced trauma. The protective effect observed in this study provides some mechanistic evidence for why indigenous people of Southeast Asia found it useful for treating kidney ailments as well as being used as a food additive.
